# How ecosystem services are changing: an accounting application at the EU level

**DOI:** 10.1016/j.ecoser.2019.101044

**Published:** 2019-12

**Authors:** Sara Vallecillo, Alessandra La Notte, Silvia Ferrini, Joachim Maes

**Affiliations:** a European Commission – Joint Research Centre, Ispra, Italy; b Centre for Social and Economic Research on the Global Environment (CSERGE), School of Environmental Sciences, University of East Anglia, Norwich, United Kingdom; c Department of Political and International Sciences, University of Siena, Italy

**Keywords:** Supply table, Use table, Official statistics, Spatial models, Drivers of changes, Monetary value

## Abstract

Ecosystem services accounts are a useful tool that provides relevant information on the role of ecosystems in delivering services, and the society benefiting from them. This paper presents the accounting workflow for ecosystem services at the European Union level adopted by the Knowledge Innovation Project on an Integrated system for Natural Capital and ecosystem services Accounting (KIP INCA) - a European Commission initiative. The workflow includes: 1) biophysical assessment of ecosystem services; 2) monetary valuation; and 3) compilation of accounting tables. Supply and use tables are presented for six ecosystem services assessed so far. The supply table shows woodland and forest, followed by wetlands, as the ecosystem types with the highest monetary value per unit area. Analyses of changes between 2000 and 2012 show an overall increase of the monetary value of ecosystem services, mainly due to an increase in demand for them. We also discuss advantages and disadvantages of adopting a fast-track approach, based on official statistics, in comparison to an accounting strategy based on spatial models. We propose a novel workflow for ecosystem services accounts, focused on assessment of the actual flow of ecosystem services, making a significant contribution to further development of the technical recommendations for ecosystem services accounts.

## Highlights

• We accounted for ecosystem services using methods with levels of complexity.
• Provisioning services refer to the ecosystem contribution (not to human inputs).
• Spatial models are useful to quantify the use of the service (actual flow).
• The assessment of the demand not covered by ecosystems is useful for decision making.
• The value of each ecosystem type represents the yearly flow of the ecosystem asset.

## 1 Introduction

There is an increasing need to quantify the contribution of ecosystems to human well-being and the economy (Bateman et al., 2013, European Commision, 2014). In this context, ecosystem services accounts are a useful tool that provides relevant information on the role of ecosystems in delivering services, and how they satisfy a demand set by society (Mace, 2019, United Nations, EC, FAO, OECD and World Bank, 2014b). Ecosystem services accounts systematically integrate and organise data derived from different sources, establishing a solid linkage between ecosystems and socio-economic systems. The development of consistent ecosystem accounts, aligned with the System of National Accounts (SNA), enables direct comparisons with economic indicators (Vardon et al., 2018).

The SNA is an international standard for the systematic compilation and presentation of economic data. It provides information on how much economic sectors produce, how much households consume and save, the level of investments and the amount of trading with the rest of the world. The SNA represents the entire economy in a simplified way, through being integrated and internally consistent. The role of natural capital is not yet transparently recorded in the SNA; it needs to be complemented by ecosystem accounts. The way to integrate the natural capital domain into the SNA is through satellite accounts. In satellite accounts, the core statistical framework is applied to outputs designed to meet specific/crosscutting uses not originally contemplated in the SNA. Specifically, in this case, information related to the environment is processed, framed and reported alongside the core SNA framework. The System of Environmental-Economic Accounting (SEEA) proposed and supported by the United Nations (UN) since 1993 provides methodological guidelines for setting up satellite accounts relating to natural capital. Specifically, the SEEA Experimental Ecosystem Accounting (SEEA EEA) (United Nations et al., 2014b) targets accounts reflecting the role of ecosystems and their services.

The different modules of the SEEA EEA include accounts for ecosystem extent, condition and services, and the thematic accounts. According to the SEEA EEA, ecosystem services accounts measure the supply and use of ecosystem services in physical and monetary terms, which helps to integrating the results of ecosystem accounting with other economic indicators derived from the SNA. Supply and use of ecosystem services are specific accounting terms; they refer to the amount of ecosystem service (ES) provided by ecosystems and the amount used by socio-economic systems, also termed actual flow (United Nations et al., 2014b). While ‘supply’ refers to the contribution of different ecosystem types to generate the actual flow, ‘use’ refers to the contribution of the actual ES flow to the economic sectors and households. Importantly, both the supply and use table record the actual flow, thereby respecting the ‘accounting identity’ which means that supply must equal use (La Notte et al., 2017, United Nations, EC, FAO, OECD and World Bank, 2014b).

In parallel with the progress made on the SEEA EEA, the European Union (EU) 7th Environment Action Programme and the EU Biodiversity Strategy to 2020 include objectives to develop natural capital accounts in the EU, with a focus on ecosystems and their services (European Commission, 2011). In this context, the Knowledge Innovation Project on an Integrated system for Natural Capital and ecosystem services Accounting (KIP INCA)^1^ was set up in 2016 by several services of the European Commission – Directorate-General for Environment, Directorate-General for Research and Innovation, Joint Research Centre (JRC) and European Statistical Office (Eurostat) – and the European Environment Agency. The main objective of the project is to design and implement an integrated accounting system for ecosystems and their services in the EU, by testing and further developing, the technical recommendations provided by the SEEA EEA (United Nations et al., 2014b). KIP INCA builds on the first phase of the EU initiative on Mapping and Assessment of Ecosystems and Services (MAES), which aims to map and assess ecosystems and their services in the EU. Ultimately, the key role of KIP INCA is to give support to the second phase of MAES, which focuses on the monetary valuation of ecosystem services and their integration into accounting and reporting systems by 2020.

In KIP INCA, the JRC develops, at the EU level, experimental accounts for the actual flow of a range of ecosystem services. In this paper, we present the general framework adopted by KIP INCA for ecosystem services accounts, which includes two different approaches: 1) fast-track approach based on official statistics; and 2) spatial modelling approach based on mapping of different components of ecosystem services (potential, demand, and use or actual flow). Providing a detailed explanation of the methods used to account for each ecosystem service is beyond the scope of this paper, since they can already be found in the literature (Vallecillo et al., 2018, Vallecillo et al., 2019a, Vallecillo et al., 2019b). We provide the integrated results for the six ecosystem services accounted for so far at the EU level: crop provision, timber provision, global climate regulation, crop pollination, flood control and nature-based recreation. The results for all these ecosystem services include mapping of different components of ecosystem services, supply and use tables, and an analysis of changes over time. Finally, we discuss advantages and disadvantages of the fast-track versus the spatial modelling approach to conducting the accounts for ecosystem services, also highlighting key challenges identified in the development of ecosystem services accounts at the EU level.

## 2 Accounting framework for ecosystem services accounts

Six ecosystem services accounts have been developed, including two provisioning services, three regulating and maintenance services, and one cultural ecosystem service (Table 1; Annex 2). In addition to this classification^2^ of ES, it is possible to further conceptualise the different role played by ecosystems in delivering services: they can in fact act as source (of biomass) or sink (of pollutants); they could reduce the magnitude of flows (as flood control) or provide information (as cultural ES). This additional typology proves to be useful from the accounting perspective as shown in La Notte et al. (2019b) (see Section 2.1.2.1).

**Table 1:** Description of the ecosystem services accounts currently developed.

Ecosystem services [role of the ecosystem^a^]	Definition	Years	Accounting approach	Monetary valuation method
PROVISIONING				
Crop provision [source: productivity^b^]	Ecological contribution to the growth of cultivated crops that can be harvested and used as raw material	2000, 2006, 2012	Fast-track (disentangling ecosystem contribution)	Market values
Timber provision [source: productivity^b^]	Ecological contribution to the growth of timber that can be harvested and used as raw material	2000, 2006, 2012	Fast-track (disentangling ecosystem contribution)	Market values

REGULATING AND MAINTENANCE				
Global climate regulation [sink^c^]	Sequestration of greenhouse gases from the atmosphere by ecosystems	2000, 2006, 2012	Fast-track	Carbon rates
Flood control [buffer^d^]	Regulation of runoff by ecosystems that mitigates or prevents potential damage to economic assets (i.e., infrastructure, agriculture) and human lives	2006 and 2012	Spatial model	Avoided damage cost
Crop pollination [source: suitability^e^]	Fertilisation of crops by insects and other animals that maintains or increases crop production	2000, 2006, 2012^g^	Spatial model	Market values

CULTURAL				
Nature-based recreation [information^f^]	Biophysical characteristics or qualities of ecosystems that are viewed, observed, experienced or enjoyed in a passive, or active, way by people on a daily basis.	2000 and 2012	Spatial model	Zonal travel cost method

Source: Vallecillo et al., 2019b, Vallecillo et al., 2018, Vallecillo et al., 2019a.

a Typology of ecosystem flow according to the role of ecosystems (La Notte et al., 2019b).

b 'Source: productivity' refers to the net delivery of biomass or energy eventually leaving the ecosystem.

c 'Sink' refers to the matter or energy absorbed by the ecosystem.

d 'Buffer' refers to the matter or energy flowing through the ecosystem.

e 'Source: suitability' refers to the delivery of biomass and energy generated within the ecosystem.

f 'Information' refers to the information delivered by the ecosystem (this delivery process does not modify the original state of the ecosystem).

g For 2012 the demand for crop pollination is assumed to be the same as in 2006.

Ecosystem services accounts are provided for the years 2000, 2006 and 2012, matching the years in which CORINE land cover (CLC) maps are available. For consistency with the ecosystem extent accounts carried out by the European Environment Agency, the accounting layers of CLC are used as reference data (European Environment Agency, 2006).

Data gaps hampered development of the accounts for all targeted years (Table 1). Where data were not available for a given year, we interpolated the results to fill the gap, assuming a constant trend, and made an estimate of ES accounts over time based on the best available data at the EU level.

**Fig. 1:**
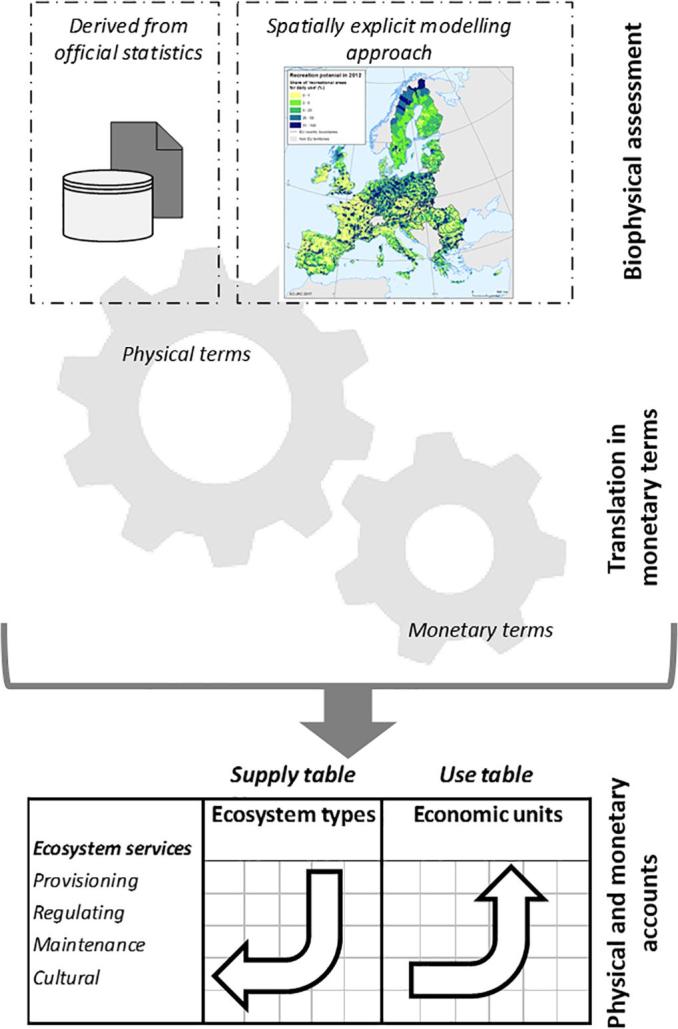
Framework adopted in KIP INCA for ecosystem services accounts.

The KIP INCA framework for ES accounts follows three main steps (Fig. 1): 1) biophysical assessment of the ecosystem service (Section 2.1); 2) translation into monetary terms (Section 2.2); and 3) compilation of supply and use tables (Section 2.3). This framework is consistent with the accounting structure of the SNA and SEEA EEA (United Nations et al., 2014b).

### 2.1 Approaches to assessment of the actual flow

ES accounts is focused on quantifying the actual flow, which is the amount of ecosystem service effectively delivered from ecosystems to socio-economic systems (Maes et al., 2013, Villamagna et al., 2013). There are many ways to quantify the actual flow, with a varying degree of complexity: approaches can range from direct use of available data, to intermediate processing and modelling. Of course, different approaches require different procedures, different expertise, and different processing time. This study shows a full range of examples.

More specifically, for some ecosystem services, the actual flow can be quantified by adopting a fast-track approach that relies on officially reported statistics (Table 1). This is the case especially (but not only) for ecosystem services that contribute to the generation of products already reported in the SNA, such as timber and crop provision. In this way, ES accounts maintain linkage with the official statistics also considered in the SNA. The fast-track approach is further described in Section 2.1.1. For other ecosystem services, official statistics do not provide data on the actual flow, and the development of spatially explicit models becomes essential for ecosystem services accounting (Table 1).

The development of spatial models for consistent quantification of actual flow is still under debate and remains highly challenging, especially for regulating ES, due to the complexity of mechanisms generating the benefit (Sutherland et al., 2018, Villamagna et al., 2013). The modelling approach is more complex than the fast-track approach. The former is based on the modelling of spatially explicit data, which requires ad hoc expertise in Geographic Information Systems and ecological background (see Section 2.1.2), but it offers more detailed and multiscale estimates (i.e., from grid cell level, to regional and national). The latter is mainly based on the exploitation of official statistics easily downloadable from various sources. In this case, simpler estimates are produced and in a consistent way with officially accepted data.

Definition of actual flow of different ecosystem services varies broadly, depending on the way ecosystem services are perceived, how they are modelled, or the proxies used to assess them. This can largely affect the outcome of ES accounts. For this reason, the definition of actual flow is provided for each ecosystem service, to set clear limits for what is explicitly considered in the accounts (Table 2). For instance, in the case of nature-based recreation, we focused only on daily recreation as the mobility function used to calculate actual flow was calibrated for daily recreation (short distance) (Vallecillo et al., 2019b).

**Table 2:** Ecosystems services components assessed for accounting.

Ecosystem service	Components	Definition	Units
Crop provision	Actual flow	Amount of crop production attributable only to the ecosystem contribution	tonne
Timber provision	Actual flow	Amount of timber growth attributable only to the ecosystem contribution	m^3^
Global climate regulation	Actual flow	CO_2_ uptake by ecosystems	tonne
Crop pollination	Potential	Extent of areas with high pollination potential	km^2^
	Demand	Extent of pollinator-dependent crops	km^2^
	Actual flow	Yield production attributable to pollination in overlapping areas between pollination potential and demand	tonne
Flood control	Potential	Extent of areas with high runoff retention potential	km^2^
	Demand	Extent of economic assets and population in floodplains	km^2^
	Actual flow	Extent of the demand with upstream protection from the upstream ecosystems with high runoff retention potential	km^2^
Nature-based recreation	Potential	Extent of service providing areas: 'high-quality areas for daily recreation'	km^2^
	Demand	Population number	number of inhabitants
	Actual flow	Estimated visits to the 'high-quality areas for daily recreation'	number of visits

#### 2.1.1 Fast-track approach

According to UN guidelines, benefits generated by ecosystem services can already be accounted for within the SNA (SNA benefit) or outside the SNA (non-SNA benefit) in satellite accounts (United Nations, 2017). The first statement suggests that ecosystem services are directly embedded in economic products generated and traded in the market system. This is the case of crops, wood and fisheries. Being embedded in an SNA product could be at the same time a risk and an opportunity, from an ES accounting perspective: the risk is of double counting the service (if the ES valuation is added without any appropriate adaptation); the opportunity is to adopt a simplified procedure that uses official statistics and makes the accounts relatively easier.

The use of official statistics, as currently available, may be misleading since they report SNA products, which also include human inputs (e.g., machinery, labour and fertilizers). The ecosystem contribution represents one of the many inputs necessary to generate the SNA products reported in statistical databases. Consequently, it is necessary to disentangle the natural input from the human input. The disentangling procedure based on official statistics, appropriately framed and justified, represents an appealing opportunity for accounting as potentially no modelling is required, and no additional data need to be created. Practitioners would in fact use official data, and eventually combine ecological information available, to extract the amount of ES flow to fill the supply and use tables. However, this procedure should be grounded in solid assumptions to assess the ecosystem contribution: the simpler the assumptions adopted, the higher the uncertainties.

This fast-track approach can also be applied to non-SNA products, when ad hoc datasets are available with appropriate estimates of the ES actual flow. For instance, the assessment of global climate regulation was based on Land Use, Land-Use Change and Forestry (LULUCF) inventories (European Environment Agency, 2018).

There is no unique way to use a fast-track approach. In this paper, for example, the fast-track approach was used in different ways for three ES:

• Global climate regulation: official data from Eurostat, reported through LULUCF inventories on CO_2_ flows (Eurostat [env_air_gge], (European Environment Agency, 2018)) were taken as proxy for this ES and they are used without any further processing (see further details in Chapter 5 and Annex 8, Vallecillo et al. (2019a)).
• Timber provision: different sets of official data (extracted from Eurostat ‘Forest Resources’ folder, Annex 2) were used to disentangle the ecosystem contribution from the total value of the SNA product (see further details in Chapter 4 and Annex 6, Vallecillo et al. (2019a)).
• Crop provision: the fast-track approach, based on official Eurostat data (Annex 2), was used in combination with biophysical modelling to assess the ecosystem contribution in a spatially explicit way (see further details in Chapter 3 and Annex 4, in Vallecillo et al. (2019a)).

#### 2.1.2 Spatial models of ecosystem services

**Fig. 2:**
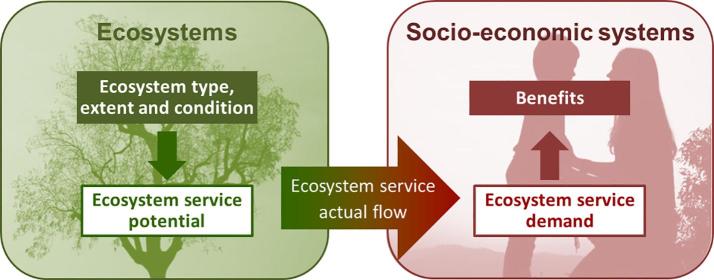
Different components of ecosystem services determining the quantity of the actual flow for ecosystem service account.

The use of spatial models to quantify the ES actual flow in KIP INCA is based on the assessment of key components that determine their flow. These components are: 1) the ecosystem service potential^3^, which is the service that ecosystems can potentially provide depending on their type, extent and condition, and 2) the ecosystem service demand, which is considered in this study as the need for a given ecosystem service by socio-economic systems (i.e. economic sectors and households) (Fig. 2; Table 2). It is important to define that the ES demand is understood as the desired levels of ES as defined by Wolff et al. (2015).

Most efforts in ecosystem services assessment have been dedicated to mapping ES potential, with significant progress in the development of mapping methods and tools (Burkhard and Maes, 2017, Maes et al., 2012, Sutherland et al., 2018). Mapping ES potential is necessary but not sufficient to determine the actual flow, which is required for ES accounts. A step forward in assessing the actual flow is the mapping of demand by socio-economic systems, which is currently gaining much more attention (Verhagen et al., 2017, Wolff et al., 2015). Certainly, the actual flow of an ecosystem service is only generated when there is demand for it (Hein et al., 2016, La Notte et al., 2019b, Maes et al., 2013).

Ultimately, the ES potential, the ES demand and their spatial relationship will determine the amount of actual flow mobilised from ecosystems to socio-economic systems to generate the benefit. The spatial relationship between ES potential and ES demand is usually more complex than a simple overlay. This relationship can be perfectly accounted for using the spatial delineation for ‘Service Providing Areas’ (SPA) (Costanza, 2008, Fisher et al., 2009, Syrbe and Walz, 2012) and ‘Service Demanding Areas’ (SDA) (Orta Ortiz and Geneletti, 2018, Schirpke et al., 2019). For the sake of simplicity, we have considered SPA as the basis area for service provision (Syrbe & Walz, 2012) where ES potential is high. This choice is made for pragmatic reasons allowing a better application of the landscape approach where SPA and SBA are spatially delineated. If areas with low ES potential were also included, practically the whole territory would be defined as SPA, which would make the modelling approach still more complex. SDA delineate the areas where the service is needed, which could be considered as Service Benefiting Areas, but only when the actual flow is generated by ecosystems and thus, producing a benefit.

In this paper, we provide examples for three ecosystem services, each having a different type of spatial relationship between SPA and SDA (Costanza, 2008). Crop pollination is an ecosystem service usually considered as ‘local proximal’, since the connection between SPA and SDA depends on proximity. However, the role of proximity was not relevant at the spatial resolution of our analyses (1 km × 1 km), since the foraging range of pollinators is smaller than the spatial scale considered. Therefore, given the resolution for our assessment, we considered the overlap as the spatial relationship between SPA and SDA, similarly to other studies (Lautenbach et al., 2012, Polce et al., 2014). In the case of flood control, the spatial relationship is ‘directional slope dependent’. This means that the actual flow of flood control (in the sense of runoff retention, Table 2) as an ecosystem service will only be generated if ecosystems providing flood control are upstream from areas demanding it, or on site. Lastly, nature-based recreation (NBR) is classified as ‘user movement related’, since the use of suitable areas for NBR will depend on the flow of people visiting them. To cope with this spatial relationship, we applied a mobility function to estimate the number of visits (Vallecillo et al., 2019b).

We claim that the spatial modelling approach proposed in this paper to quantify the actual flow is especially suitable for accounting as it helps to establish linkage between the biophysical models and the accounting tables. On the one hand, the modelled ecosystem service potential provides information about the role of different ecosystem types as providers of the service. The role of each ecosystem type will then be reported in the supply table. In some cases, the actual flow does not provide information about the contribution of each ecosystem type in delivering the service. For instance, in the case nature-based recreation the estimated number of visits obtained from the model does not tell us which ecosystem type is more visited. In this case, allocation of actual flow to the different ecosystem types is conducted *ex post*, in proportion to the different ES potential that each ecosystem presents (this issue is further discussed in Section 2.3). On the other hand, assessment of ES demand helps to determine the dependence of the economy from the actual flow, providing relevant information about the spatial location of economic sectors and households who rely on the ES. This step is useful to establish the linkage of economic units in the use table (Fig. 1), supporting the allocation of the ecosystem service to users.

##### 2.1.2.1 Where demand for ecosystem services is not satisfied

The spatial modelling of the different ES components enables assessment of another complementary indicator of ecosystem services: unmet demand (sometimes termed as ES deficit, Bommarco et al. (2013)). We consider as ‘unmet demand’ the need by society for a specific ecosystem service, which is not satisfied by ecosystems. The concept of unmet demand, although not required to quantify the actual flow, is gaining increasing attention as it provides relevant information related to ES (Haase et al., 2014, La Notte et al., 2019b, Syrbe and Grunewald, 2017). Mapping unmet demand identifies areas where ecosystem restoration may enhance the contribution of ecosystems to human well-being Section 2.1.2.1as a new supportive tool of land planning policies.

The unmet demand is therefore the result of having higher ES demand than ES potential. Mismatches between ES potential and demand may differ depending on the role of ecosystems delivering services, namely: source-productivity, source-suitability, sink, buffer or information (La Notte et al., 2019b) (Table 1). It is important to understand the role of ecosystems in delivering services, as it provides a key to interpreting (and accounting) sustainability issues that each ES presents. As shown in Table 1, the role of ecosystems in delivering services depends not only on the ES group (provisioning, regulating and maintenance, cultural), but also within the same group: regulating and maintenance services in Table 1 include ‘sink’, ‘buffer’ and ‘source-suitability’. For source-productivity and sink services, ES can be overused during the accounting period (the yearly use of the service exceeds the capacity of the ecosystem) at the expenses of the ecosystem integrity giving rise to degradation.

**Fig. 3:**
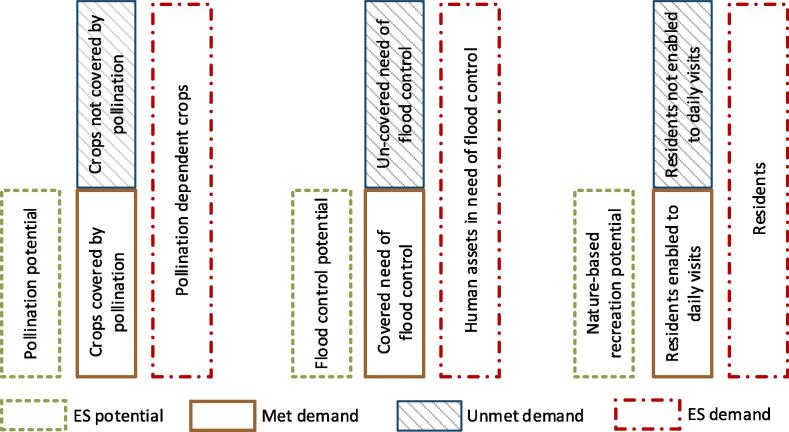
Simplified delineation of unmet demand for crop pollination, flood control and nature-based recreation. Adapted from La Notte et al. (2019b).

For other ES (e.g. ‘source-suitability’, ‘buffer’, ‘information’), overuse (when occurring) modifies the initial condition for ES potential, and thus an unmet demand is generated: i.e. when land cover and use, as initial setting for the accounting period, do not meet current needs from users during the accounting period (Fig. 3). In this paper, three ES accounts derived for the EU allow spatial assessment of unmet demand (in line with the role of ecosystems reported in Table 1):

• Crop pollination (source-suitability)
• Flood control (buffer)
• Nature-based recreation (information)

### 2.2 Translation into monetary terms

Monetary valuation of ES can be done using different approaches and valuation techniques. Since ES accounts represent satellite accounts for the SNA, their valuation should be in line with the exchange value principles applied in the core accounting system (United Nations et al., 2014b). In this way, all monetary valuation methods applied in this paper are consistent with the SNA. This implies that all valuation techniques based on stated preferences (e.g. contingent valuation) should not be applied, unless justified for consistency with the SNA transaction prices. Moreover, restoration costs that consider the whole ecosystem are not appropriate for ES flows due to the different perspective, i.e. holistic versus one-by-one assessment of ecosystem service flows.

In our approach, we applied valuation techniques consistent with SEEA EEA recommendations (Table 6.1 in United Nations (2017)), namely: market values, carbon rates, avoided damage cost and travel cost method (Table 1). We term monetary valuation as ‘translation into monetary terms’ to highlight the leading role of biophysical assessment in the monetisation of ES (Fig. 1). The purpose of monetary valuation is to represent the outcome of the biophysical assessment, using a common unit allowing aggregation among ES and linkage with SNA economic accounts. As with the biophysical assessment, we can also experience different levels of complexity in the monetary valuation (Table 1).

When a fast-track approach is applied, the monetary valuation is straightforward: since official datasets are available, once the assessment is available in physical terms, it is multiplied by a unit currency value (as in the case of crop and timber provision and crop pollination, all based on Eurostat datasets). The same approach is also applied to global climate regulation, where high-level institutions provide estimates of effective carbon rates (OECD, 2016).

When biophysical modelling is used, the procedure for monetary valuation is more complex. There should be a direct connection between: (i) any change that occurs in the key variables (e.g. proximity of people to ‘high-quality areas for daily recreation’) ruling the biophysical model; (ii) outcomes obtained in biophysical terms; and (iii) their valuation in monetary terms. Biophysical assessment cannot be ancillary information in the monetary valuation, but (as previously stated) the leading driver of the outcome in monetary terms.

For some ES, such as flood control and nature-based recreation, a model is built for the monetary valuation. For flood control, estimates based on avoided damage costs are undertaken using a complex spatial processing procedure (Vallecillo et al., under review). For nature-based recreation, a zonal travel cost method and a mobility function are applied.

Given the experimental nature of these ES accounts, methods applied are subject to readjustments that may result in substantial changes to the values reported. For the purpose of this paper, we have applied some updates in accounting for crop pollination. In Vallecillo et al. (2018), the actual flow of crop pollination was calculated by developing spatial models that allow estimation of the percentage of yield that is attributable to crop pollination (i.e. crop pollination contribution), based on CAPRI data (Britz & Witzke, 2014). The crop pollination contribution was then directly applied to Eurostat economic accounts (Eurostat [aact_eaa02]). For instance, if the economic value for a given year was EUR 10 000 and the crop pollination contribution was 30 %, then the actual flow in monetary terms would be EUR 3 000. This allows compilation of the accounting tables in monetary terms, but not in biophysical units. To solve this issue, we present in this paper a more comprehensive approach by: 1) applying the crop pollination contribution to Eurostat data in physical terms (tonne [apro-crop], Annex 2); and 2) multiplying the physical units by a unit euro value also provided by Eurostat ([aact_uv01], Annex 2). We consider that this updated approach ensures greater consistency with other ES accounts, where this same procedure is applied: accounts in physical and monetary terms thus run in parallel.

In this application, all monetary estimates are kept constant over time. This choice is justified by the purpose of highlighting changes in biophysical assessment in the trend over time. Future updates of ES accounts will apply a standard procedure to account for change in monetary values in time series, more in line with the common practice of national accounts.

### 2.3 Accounting tables

Supply and use tables represent the accounting format for ES. Accounting tables can be built in physical or monetary terms (Fig. 1). In the SNA, supply tables report goods and services produced by each economic sector. In ecosystem services satellite accounts, the supply table shows the flow of each service provided by different ecosystem types. For KIP INCA, we are following the MAES ecosystem classification (Maes et al., 2012). The introduction of ecosystem types as additional institutional sectors enlarges the scope of the production boundary. This extension of SNA considers ES flow not as passive inputs into the economic transactions but rather as active mechanism impacting and impacted by economic sectors and households (La Notte et al., 2019b).

In the SNA, the use tables show the allocation of goods and services by economic sectors as intermediate consumption (i.e. used by other industries in the production of their output), and as final consumption (i.e. purchases of each product by each category of final user – households, government, export). In ES satellite accounts, the use table shows the flow of each ecosystem service to different users (termed ‘economic units’ in accounting, which include economic sectors or households). The structure of the use table remains consistent with the SNA, and uses as definition of economic sectors the Statistical Classification of Economic Activities in the European Community (NACE Rev. 2). Moreover, global society needs to be added as user of global ecosystem services such as global climate regulation^4^.

For some ES, the quantitative allocation to ecosystem types in the supply table, and to economic sectors and households in the use table, is already clear from the description of the service or from the structure of the biophysical model, e.g. cropland for crop provision, forest available for wood supply within woodland and forest for timber provision. For other ecosystem services (i.e. nature-based recreation and flood control; see detailed citation in Annex 2), *ex post* processing is required to allocate the actual flow to the ecosystem types, considering their different potential to deliver the service (e.g. forests retain more runoff than shrubland). Since ecosystem service potential is a function of ecosystem extent and condition (Maes et al., 2013; Fig. 2), we calculated the weighted potential for each ecosystem type (Eq. (1)).

(1) $$\mathrm{Weighted}\;potential=C_{f}\times Relative\;extent$$

where *C_f_* is a correction factor for each ecosystem type that considers the parameters set in the spatial model, where the role of different ecosystem types in providing a given service is taken into account (as a proxy for ecosystem condition to provide the service). For instance, the role of forest in providing recreation opportunities and flood control is higher than the role of shrubland. It follows that forest in the spatial model receives a higher score than shrubland, as confirmed by the literature and/or expert opinion. Scores assigned to the different classes of land cover are then averaged for the MAES ecosystem types (Maes et al., 2013), which are then used as a correction factor (*C_f_*). The relative extent of each ecosystem type was calculated as the ratio between the extent of the ecosystem in the Service Providing Areas and the total extent of SPA. The ‘weighted potential’ accounts for the variability in the role of each ecosystem type in delivering the service, also considering the extent or amount of each ecosystem type. For instance, 1 km^2^ of forest retains more runoff than 1 km^2^ of shrubland, but 10 km^2^ of shrubland would retain more runoff than 1 km^2^ of forest. The weighted potential (i.e. extent multiplied by the correction factor) is then used to allocate the total actual flow, in relative proportion to the values obtained. In other words, if the weighted potential of forest is 10 times larger than of shrubland, the amount of actual flow allocated to forest will also be 10 times larger.

Allocation of the actual flow in the use table based on demand is more straightforward, especially for some ecosystem services. When dealing with provisioning services, the SNA product generated only by the ecosystem already has a recipient: these services provide input to economic sectors to produce such as agriculture and forestry. It is a similar case for crop pollination, since the benefit generated is also an SNA product used by the agricultural sector (also mapped as demand for crop pollination: pollinator-dependent crops). In fact, mapping ES demand provides the information required to allocate the actual flow to economic units in the use table. People exert a demand for nature-based recreation, becoming the users of this ecosystem service and therefore the actual flow is allocated to households in the use table. In the case of flood control, the actual flow is only calculated where there is demand for this ecosystem service: economic assets such as artificial and agricultural areas. Thus, we can directly calculate the actual flow separately for each land cover type delineating the demand, and establishing correspondence with the different economic sectors. For instance, the actual flow of flood control generated in urban fabric is allocated to households, while the actual flow being used in agricultural areas is allocated to the agriculture sector (Vallecillo et al., 2019a, Vallecillo, under review).

In the case of global climate regulation, the actual flow is usually allocated to global society since it is considered as a global ES (Costanza, 2008) and the whole society benefits from the mitigation of CO_2_ emissions. However, allocation of the actual flow for this ES might go beyond the final beneficiaries and focus on the emitters of CO_2_ (Morri et al., 2014). By doing so, the actual flow in the use table could potentially be allocated to the actors that determine to what extent the use of this ES is needed (e.g. emitters), making it more relevant to decision-making (Wolff et al., 2015). In fact, legal obligations are set by political institutions to reduce CO_2_ emissions, and thus it becomes necessary for society to look at the drivers of pressure that ultimately makes this ecosystem service necessary for the society.

When using the accounting terminology defining ‘assets’, we need to point out that in our approach, the asset is not mixed with the concept of resources, but refers to ecosystem types. For example, standing timber is a natural resource that can be measured as opening and closing stock for each accounting year. When it comes to ES accounts, we look at woodland and forest that provides not only timber, but also climate regulation, flood control, recreation and so on. The ‘asset’ in an ecosystem accounting perspective should thus refer to the capacity of the ecosystem type woodland and forest to provide a range of ecosystem services, and should not be confused or mixed with the stock of standing timber (which is a resource). Although there is still discussion on how to clearly frame the notion of asset in ES accounts, one attempt that puts together ecosystem types and ES and compares this new approach to the traditional resource accounts is proposed in La Notte et al. (2019a). The risk in mixing ecosystem types and resources would be the inconsistency with the overall SEEA framework, since natural assets are already accounted for in the Central Framework (United Nations et al., 2014a).

## 3 Accounting results and discussion

### 3.1 Mapping the actual flow of ecosystem services

**Fig. 4:**
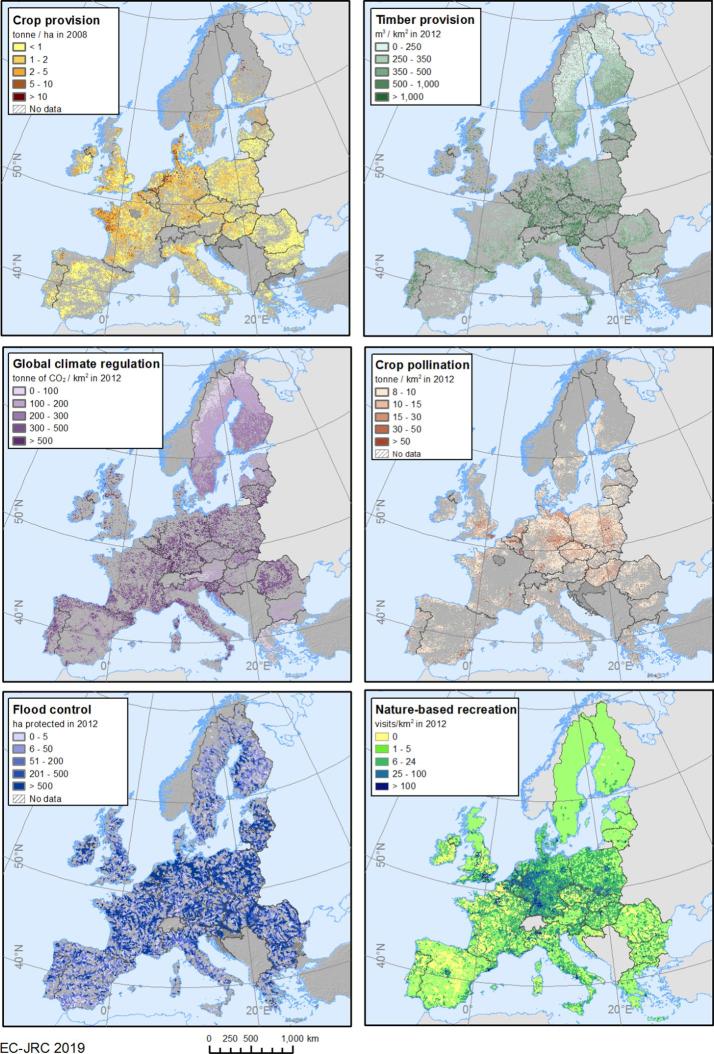
Maps of the actual flow of six ecosystem services.

Maps of the actual flow of ecosystem services show differences among their spatial patterns (Fig. 4). For ecosystem services assessed through the fast-track approach (i.e. crop and timber provision, and global climate regulation), the spatial pattern is highly driven by the productivity of ecosystems in terms of biomass growth. Central Europe, covering Continental and Atlantic biogeographic regions, shows higher values for crop provision, timber provision and global climate regulation. In these regions, with high precipitation and mild temperatures, ecosystem productivity is higher than in the Mediterranean, Alpine and Boreal regions, where biomass growth is limited by water scarcity in the Mediterranean region and by a short growing season in the Alpine and Boreal regions. Importantly, the actual flow of these three ecosystem services is mapped only in areas where the land cover map (CLC) shows agricultural land cover for crop provision, and forest cover for timber provision and global climate regulation. These land covers are taken into account in allocating the actual flow in the use table to agricultural and forestry sectors, respectively.

For ecosystem services for which actual use is derived from spatial models, the spatial distribution of the actual flow largely depends on demand. For instance, the actual flow of crop pollination is only generated in those areas where farmers decide to grow pollinator-dependent crops – according to the Common Agricultural Policy Regionalised (CAPRI) model (Britz & Witzke, 2014) and consistent with the distribution of agricultural areas in the land cover map– but also where the environment is suitable for pollinators (i.e. presence of SPA). In the case of flood control, the actual flow is only found where economic assets (built-up areas, infrastructure, and agricultural areas) are located in floodplains, but also where ecosystems located upstream from the demand contribute to reducing runoff. For visualization purposes, the actual flow for flood control has been aggregated at sub-catchment level, as the extent of demand areas is too small for being appreciated on the map.

Similarly, nature-based recreation shows high values for actual flow where there is more abundant population making use of suitable nearby areas for daily recreation (central Europe and capital cities). This pattern is due to the way the actual flow was quantified. For this experimental account, nature-based recreation for households covers only one part of the numerous recreation flows that could be quantified (such as the contribution of nature to the tourism sector). In this case, the actual flow of nature-based recreation estimates only visits to high-quality areas on a daily basis (i.e. short distance recreation, walk after work). As such, visits are quantified only where population lives less than 4 km from these high-quality areas (SPA). Therefore, municipalities (used as spatial reference unit for this model) with a high number of inhabitants and SPA closer than 4 km show higher actual flow. Since we considered daily recreation, high-quality areas that are beyond 4 km from population centres would not be suitable for daily activity, so would not contribute to generating actual flow (see Vallecillo et al., 2019b for further details).

#### 3.1.1 Unmet demand

**Fig. 5:**
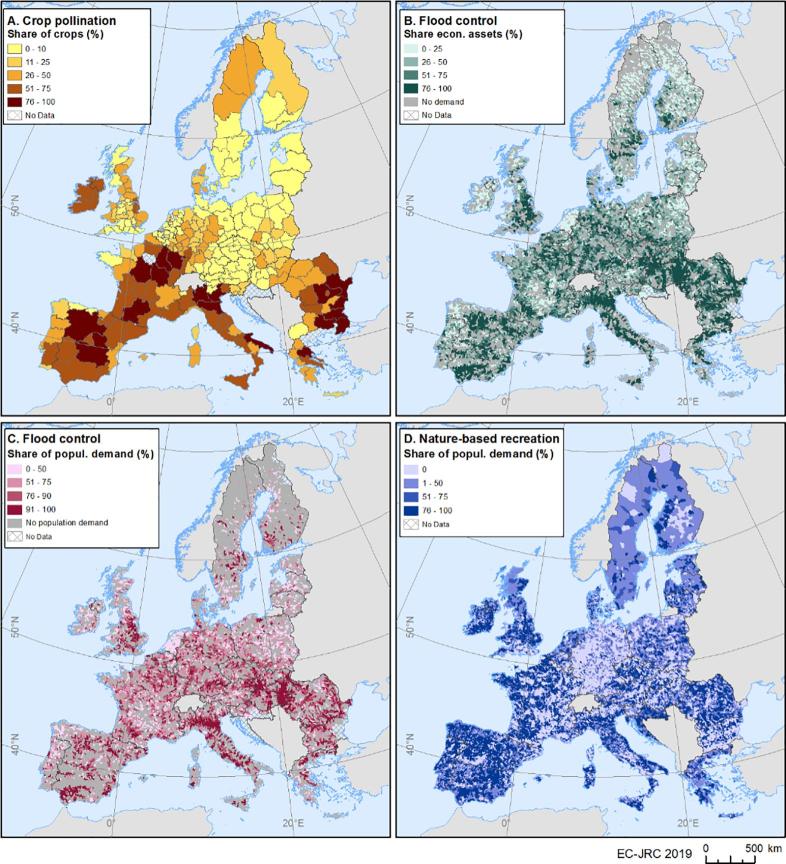
Unmet demand for three ecosystem services in 2012.

Maps of unmet demand, represented as the percentage of demand not satisfied by ecosystems, can support land planning and ecosystem restoration policies showing the patterns where needs not met are higher (Fig. 5). For instance, for crop pollination there is a very low percentage of unmet demand in north and central Europe, due to high environmental suitability to support pollinators (bumblebees and solitary bees) in these areas. Regions with higher unmet demand for crop pollination should be prioritised for restoration of pollinator-friendly habitats, which would contribute to enhancing the ecosystem contribution to human well-being. The unmet demand for flood control, quantified at sub-catchment level, has been assessed both for economic assets and for population in floodplains. Higher values of unmet demand are mainly found in arable plains and large urban areas where the ES potential is generally low. In the case of the Netherlands, the level of protection by artificial defence measures is high enough to safeguard all economic assets from flooding for the maximum return period considered (500 years). Therefore, we assumed that in this country, the demand for flood control is fully satisfied by the current level of protection and thus the unmet demand is considered to be zero. In the case of nature-based recreation, as mentioned above, we considered the estimated visits on a daily basis to ‘high-quality areas’ (i.e. assuming a minimum proximity of 4 km between these areas and the inhabitants in a given area). As a result, countries such as Germany have low unmet demand because of the proximity of ‘high-quality areas’ to urban centres. By contrast, other regions – such as some municipalities in Finland – show very high unmet demand. Although there are not very large urban areas there, a high share of the population lives further away from ‘high-quality areas’, since these cities are mainly surrounded by arable land, which offers low nature-based recreation opportunities.

### 3.2 Accounting tables in monetary terms

The accounting results show nature-based recreation (NBR) as the ecosystem service with the highest monetary value, representing 40 % of the total monetary value of the six ES accounted for at the EU level (Table 3).

**Table 3:** Supply table in absolute terms for six ecosystem services in 2012.

This result is in line with other studies (Remme et al., 2015, Sunderland et al., 2018) which found that NBR represents more than 30 % of the annual monetary value of ecosystem services. The relative value of this ES remains one of highest, despite the different approaches to monetary valuation used.

In the case of Sunderland et al. (2018), the monetary value reported for NBR in natural reserves in the United Kingdom is about EUR 25 million per year, while our estimates for the same country are about EUR 4 billion (Vallecillo et al., 2019a). The lower estimates by Sunderland et al. (2018) can be explained by them focusing their attention on natural reserves, ignoring many locally accessible sites that people enjoy on a daily basis.

In relative terms, crop pollination represents 8 % of the total value. This is because we account here for the share of crop production attributable to pollination only in areas with high environmental suitability for pollinators (i.e. in service providing areas), and where there is demand (i.e. pollination dependent crops). Pollination is also reported by de Groot et al. (2012) as the ecosystem service with the lowest relative value in monetary terms among the ES presented in this paper. Despite having the lowest relative importance, its annual value in 2012 reached approximately EUR 10 billion, which represents about 20 % of the total yield value of pollination-dependent crops.

The supply table relative to the ecosystem extent may be useful for comparing findings across other accounting case studies. Table 4 (last row) shows the ‘asset’ woodland and forest as the ecosystem type providing the highest monetary value per unit area per year, followed by wetlands and sparsely vegetated land.

**Table 4:** Supply table in relative terms for six ecosystem services in 2012.

This finding is not surprising, since these ecosystems are usually recognised in the literature as key ecosystems for delivery of services (Burkhard et al., 2014, Navarro and Pereira, 2015, Rodríguez-Loinaz et al., 2015). In the case of the ‘asset’ wetlands, although absolute monetary values per year are not as high as for woodland and forest, the reduced extent of this ecosystem type makes them valuable per unit area. In fact, this monetary value could be significantly enhanced if appropriate management practices and rewetting are implemented, to favour the natural functioning of these ecosystems as CO_2_ sinks (Nahlik & Fennessy, 2016).

By contrast, the ‘asset’ urban ecosystem shows the lowest monetary value per year. This value is certainly not negligible, given that most of the extent of urban ecosystems is built-up land covers (i.e. residential areas, industrial and commercial areas; Annex 1) with no ES delivery. Therefore, the monetary value reported in the accounts is mainly due to the presence of green urban areas, which is the key land cover type acting as supplier of ecosystem services in urban ecosystems, as defined in the MAES ecosystem classification (Annex 1). If we considered just the extent of green urban areas, we would obtain a relative value for urban ecosystems of EUR/km^2^ 56 000, which is even higher than the value of forest.

Finally, the monetary value reported per year for the ‘asset’ rivers and lakes and coastal areas should be considered with caution, since it was only assessed for nature-based recreation and the full range of services provided by these ecosystems is not properly represented.

The use table shows households as the main users of ecosystem services, mainly due to the high monetary value of nature-based recreation (Table 5). A higher monetary value than for other sectors can also be explained by the fact that in the case of NBR, households are the final consumers: no further processing, transformation, or trading takes place. For all other ES, there might be intermediate consumption and further processing, which may increase the value added by orders of magnitude.

**Table 5:** Use table for six ecosystem services in 2012.

The agricultural sector is the second most important user, mainly for crop provision and crop pollination. We should bear in mind that the use table represents the entry point into the SNA. The agricultural sector can generate products for intermediate and final consumption by other economic sectors, both for domestic economy and international trade. The food industry is one of the key determinant sectors for the wealth of any country. Agriculture can produce raw crops thanks not only to farmer input but also to ecosystem contribution. The yearly flow of yield (from crop provision and pollination) and protection (from flood control) that the agricultural sector receives from ecosystems is estimated at EUR 31 billion. Under a scenario of reduced ES flows to agriculture, the production of raw crops (and all the transformation and trading activities that depend on them) would be more expensive to farmers.

### 3.3 Changes over time

The accounting of ecosystem services over time shows an increase in monetary value in the actual flow of ecosystem services assessed at the EU level, except for timber provision (Table 6). However, this increase is not necessarily driven by an enhancement of the natural capital. For a proper understanding of the drivers behind changes in the actual flow, we should consider the interaction of ecosystem potential and demand, which are only available for ES accounted for through spatial models (Table 6).

**Table 6:** Summary table at the EU level of components of ecosystem services assessed.

In this context, the increase in the monetary value of nature-based recreation is mainly explained by an important increase in ES potential, but also by increased demand from population in need of that service (Table 6). On the other hand, the increase in the monetary value of crop pollination is mainly due to the expansion of pollinator-dependent crops (i.e. ES demand), given that pollination potential decreased on average at the EU level. Of course, the final impact of changes in ES potential and demand will depend on the spatial relationship required between these two components.

Interpretation of the increase in monetary value of flood control by ecosystems is more complex, since at the EU level there are practically no changes in the ES potential or demand, and the actual flow in biophysical terms even decreases (Table 6). As discussed in Vallecillo et al. (under review), a more detailed analysis of the ES components shows that the increase in the monetary value of flood control is due to the sprawl of artificial areas in floodplains that benefit from runoff retention by upstream ecosystems. Indeed, urban sprawl in floodplains is taking place at the expense of agricultural areas, showing as a result a practically negligible net decrease in demand for flood control (Table 6). This replacement of agricultural areas by artificial land in floodplains explains the increase in monetary value of flood control. This is due to higher cost avoided by upstream ecosystems when artificial areas, instead of agricultural areas, are protected.

Analyses of changes in unmet demand are also useful in understanding the role of natural capital in contributing to human well-being (Table 7). Interestingly, unmet demand increases for crop pollination and flood control (except for unmet demand from agricultural land), matching those ES where potential does not change, or even slightly decreases in the case of crop pollination. On the other hand, for nature-based recreation the increase in ES potential (mainly due to designation of Natura 2000 sites) is high enough to satisfy the increasing need for this ES. In fact, the number of inhabitants considered as unmet demand (for recreation on a daily basis) decreases for the period analysed. This suggests that, in a context of increasing demand for ES, enhancement of ES potential would also be required to meet socio-economic needs, especially in those areas where demand is currently not satisfied (Fig. 4). This would result in enhancement of the monetary value of ecosystem services, and therefore the benefits generated by ecosystems to society.

**Table 7:** Unmet demand for the ecosystem services assessed through spatial models.

Although interpretation of changes over time is more difficult for ecosystem services assessed through the fast-track approach, some inference can still be drawn. The increase in the actual flow for crop provision can be interpreted as an increase in demand (Wolff et al., 2015), which is leading to an intensification or enhancement of agricultural practices, increasing biomass growth in absolute and relative terms. Although we have developed an approach to disentangle the contribution of ecosystems to biomass growth from the role of human inputs, it was not possible to apply this in a dynamic way over time and the ecosystem contribution is considered to be fixed for all years assessed. Therefore, the increase in crop provision reflects only the intensification of crop production (Eurostat, 2018), not the increase in the percentage of ecosystem contribution to producing yield.

In the case of timber provision, the increase in the actual flow in biophysical terms is less important (only +1 % in absolute terms – Table 6), which also aligns with changes in global climate regulation, since it largely depends on biomass growth in forests.

## 4 Conclusions

We have presented a novel workflow for ecosystem services accounts at the EU level, focused on different methods for assessment of the actual flow of ES. This work presents a sound methodology, to be further discussed and readjusted to contribute to experimental development of the SEEA EEA. We also provide relevant results on ecosystem services and their changes over time, based on the best data available at the EU level. Given the experimental character of these accounts, values reported here are susceptible to change in future, before the method for the accounts can be consolidated. Updating and improving of methodologies are common practices for standard accounts, which is even more evident when developing experimental accounts.

The KIP INCA project is testing implementation and development of the SEEA EEA guidelines (United Nations, 2017, United Nations, EC, FAO, OECD and World Bank, 2014b) suggested by the United Nations Statistical Division. The SEEA EEA offers a good basis for experimental testing. While accounting for the actual flow of ES, it has been possible to achieve conceptual and technical advances useful to the future applications of ecosystem services accounts. The key novelties presented in this study are: 1) accounting for the actual flow of provisioning services by excluding the role of human inputs; 2) the use of spatial models to quantify ES actual flow, based on the relationship between ES potential and ES demand; 3) the importance of correctly identifying ecosystem types as ‘assets’ providing a range of ES flows; and 4) the adoption of alternative accounting approaches depending on the data and type of ES.

The first point highlights the need to disentangle the ecosystem contribution in the generation of provisioning services (crop and timber provision) from what is generated by human inputs (e.g. fertilisers, machinery). Most ecosystem services accounts carried out so far take SNA products (crop yield and timber) as proxy for ES flow (Office for National Statistics, 2018, Ouyang et al., 2016, Remme et al., 2015). We claim that considering the biomass growth derived from human inputs as ecosystem service is conceptually inappropriate, and leads to an overestimation of ES provisioning. This may lead to misleading messages and flawed analysis of synergies and trade-offs among ecosystem services.

The second point brings attention to the suitability of spatial models for quantifying ES actual flow based on the main drivers: ES potential and ES demand. As demonstrated in our approach, the development of spatial models is suitable for accounting purposes, and it also supports connection with the supply and use tables.

The third point helps to consistently identify what is an ‘asset’ in ES accounting. It is important not to mix the role of ecosystem types (Experimental Ecosystem Accounts) with natural resources (Central Framework) in the SEEA framework. This concept is still under discussion in the SEEA EEA revision process. In our proposal, the sum of ES per ecosystem type represents the yearly flow provided by the ecosystem asset (as shown in the supply table).

The fourth point shows the potential to apply different approaches to ecosystem services accounts. The fast-track approach can mainly be applied to those ecosystem services related to biomass production, where the ecosystem service flow is more easily measurable than for other ecosystem services (e.g. regulating services). Data for these ecosystem services are more easily available in official statistics. The advantage of this fast-track approach lies in the use of available information without the need to model. The disadvantage lies in the lack of underlying information to understand the changes over time and the cause-effect relationships that should be contrasted with available literature or other complementary indicators. Furthermore, for the purpose of accounting for global climate regulation, the use of the LULUCF inventories presents some limitations. Firstly, data refer only to managed land, excluding natural (non-managed) areas. This might explain why wetlands appear as net sources of CO_2_ instead of sinks, as usually reported in the literature (Whiting and Chanton, 2001). Secondly, there needs to be a standardisation of methodologies applied across countries. This could enhance the suitability of LULUCF inventories for a regular update to account for global climate regulation.

The accounting approach based on spatial models for ES provides data about the key drivers of change in the service used: ES potential and demand. This facilitates a detailed assessment of the changes in the actual flow, helping to understand the role of ecosystems providing the service and/or the role of society benefiting from it. It also provides complementary data useful for land planning, such as the maps of unmet demand that show areas where ecosystem restoration may enhance the ecosystem contribution to human well-being. For instance, in the case of nature-based recreation, statistics on real daily use of natural areas could be gathered for the adoption of a fast-track approach. However, with this fast-track approach we would not be able to understand why changes take place and what exactly is the role of natural capital in determining the service. A drawback of the modelling approach is that it requires ad hoc expertise and these models are not easily usable or accessible for practitioners or policymakers. A solution could be to develop tools in Geographic Information Systems that include the whole accounting workflow. These tools would contribute to making ES accounts more accessible to practitioners, by taking advantage of the modelling technique developed by experts, and at the same time by using their own detailed database.

These different assessment procedures could contribute to building a more comprehensive tier approach in ES accounting, consistent with the already solid ES mapping tier approach (Burkhard & Maes, 2017) and also with valuation and accounting principles, which would constitute a novelty. The more applications become available, the more it will be possible to structure a theoretically consistent system in line with practitioners’ needs.

The development of the experimental ecosystem services accounts under KIP INCA highlights the complexity of developing these accounts, both conceptually and methodologically. In this context, there is a need to develop accounts for more ecosystem services, since each of them presents very different characteristics. ES accounts cannot be generalizable, since the assessment of each ecosystem service is very different and the accounts need to be tailored accordingly.

It is important to highlight that the accounts developed at the EU level present many challenges, mainly related to data availability: sometimes data do not exactly match the years assessed, or they are not available at the required spatial resolution. This mainly hampers the development of consistent accounts for a representative time series. Further developments should focus on obtaining better data suitable for accounting, for instance spatial distribution and production of pollinator-dependent crops over time for crop pollination, and temporal series for high natural value of farmland and forest for nature-based recreation.

Despite the issues with data availability, the methods developed under KIP INCA are suitable for application to different geographic areas and at different spatial scales. Currently, there are a limited number of examples of ES accounts (e.g. Hein et al., forthcoming, Keith et al., 2017, Office for National Statistics, 2018, Campos et al., 2019) and more experimental exercises would be needed to compare the results of applying different methods or input data at different spatial scales.

ES accounts constitute a step forward in ES mapping and reporting of indicators. In this sense, ES accounts presented in this paper are the starting point to further develop a system that is truly integrated with economic accounts. In fact, integration with the SNA is essential for ES accounts to play a role in economic modelling, analysis and planning. Future releases under KIP INCA will present accounts for water purification, soil erosion control and habitat maintenance. The addition of new ecosystem services to the accounting tables may result in significant changes in the monetary value of ‘assets’ and the relative importance of each ecosystem type. Further efforts should be made in accounting for a comprehensive list of ecosystem services, allowing robust analysis of synergies and trade-off between them.

## Figures and Tables

**Table:** 

MAES ecosystem	CORINE land cover
Urban	Continuous urban fabric
	Discontinuous urban fabric
	Industrial or commercial units
	Road and rail networks and associated land
	Port areas
	Airports
	Mineral extraction sites
	Dump sites
	Construction sites
	Green urban areas
	Sport and leisure facilities
Cropland	Non-irrigated arable land
	Permanently irrigated land
	Rice fields
	Vineyards
	Fruit trees and berry plantations
	Olive groves
	Annual crops associated with permanent crops
	Complex cultivation patterns
	Land principally occupied by agriculture, with significant areas of natural vegetation
	Agro-forestry areas
Grassland	Natural grasslands
	Pastures
Heathland and shrub	Moors and heathland
	Sclerophyllous vegetation
Woodland and forest	Broad-leaved forest
	Coniferous forest
	Mixed forest
	Transitional woodland-shrub
Sparsely vegetated land	Beaches, dunes, sands
	Bare rocks
	Sparsely vegetated areas
	Burnt areas
	Glaciers and perpetual snow
Wetland	Inland marshes
	Peat bogs
Rivers and lakes	Water courses
	Water bodies
Marine inlets and transitional water	Salt marshes
	Salines
	Intertidal flats
	Coastal lagoons
	Estuaries

**Table:** 

	Data sources	Links/References	Spatial coverage
PROVISIONING			
Crop provision	Source: Vallecillo et al. 2019a, page 13		
ECOSYSTEM CONTRIBUTION	Data derived from an emergy-based approach from Pérez-Soba et al. 2019	JRC Report (https://ec.europa.eu/jrc/en/publication/emergy-perspective-natural-and-anthropic-energy-flows-agricultural-biomass-production)	EU-25 (lack of data for Cyprus, Malta and Croatia)
YIELD DATA (biophysical terms)	EUROSTAT [apro_cpsh1]	Eurostat dataset (https://appsso.eurostat.ec.europa.eu/nui/show.do?dataset=apro_cpsh1&lang=en)	
MONETARY VALUE	EUROSTAT [aact_uv01]	Eurostat dataset (https://appsso.eurostat.ec.europa.eu/nui/show.do?dataset=aact_uv01&lang=en)	
**Timber provision**	Source: Vallecillo et al. 2019a, page 27		
ECOSYSTEM CONTRIBUTION	EUROSTAT [naio_10_cp16]	Eurostat dataset (https://appsso.eurostat.ec.europa.eu/nui/show.do?dataset=naio_10_cp16&lang=en)	EU-27 (excluding Malta: no forestry sector)
YIELD DATA (biophysical terms)	EUROSTAT [for_vol_efa] [for_vol]	Eurostat datasets (https://appsso.eurostat.ec.europa.eu/nui/show.do?dataset=for_vol_efa&lang=en; https://appsso.eurostat.ec.europa.eu/nui/show.do?dataset=for_vol&lang=en)	
MONETARY VALUE	EUROSTAT [for_vol_efa] [for_eco_cp]	Eurostat datasets (https://appsso.eurostat.ec.europa.eu/nui/show.do?dataset=for_eco_cp&lang=en; https://appsso.eurostat.ec.europa.eu/nui/show.do?dataset=for_vol_efa&lang=en)	

REGULATING AND MAINTENANCE			
Global climate regulation	Source: Vallecillo et al. 2019a, page 42		
ACTUAL FLOW	EUROSTAT [env_air_gge] (EEA, 2018)	Eurostat dataset (https://appsso.eurostat.ec.europa.eu/nui/show.do?dataset=env_air_gge&lang=en)	EU-28
MONETARY VALUE	C rates based on OECD (2016)	OECD report (https://www.oecd-ilibrary.org/taxation/effective-carbon-rates_9789264260115-en)	
**Flood control**	Source: Vallecillo et al. 2019a, page 67		
FLOOD CONTROL POTENTIAL	Land-use land cover data	CORINE Accounting Layers (https://www.eea.europa.eu/data-and-maps/data/corine-land-cover-accounting-layers#tab-european-data)	Data available for EU-26 (excluding Cyprus and Malta, and some regions in Croatia, Bulgaria and Finland)
	Slope	EU Dem 100 m: https://land.copernicus.eu/pan-european/satellite-derived-products/eu-dem/eu-dem-v1-0-and-derived-products/eu-dem-v1.0?tab=download	
	Hydraulic properties	USDA soil textural classes: https://esdac.jrc.ec.europa.eu/resource-type/datasets	
	Imperviousness	https://land.copernicus.eu/pan-european/high-resolution-layers/imperviousness/view	
	Riparian zones	https://land.copernicus.eu/local/riparian-zones	
DEMAND FOR FLOOD CONTROL	Economic assets (agricultural and artificial)	CORINE Accounting Layers (https://www.eea.europa.eu/data-and-maps/data/corine-land-cover-accounting-layers#tab-european-data)	
	Flood hazard map (return period 500 years)	https://data.jrc.ec.europa.eu/collection/id-0054	
	Road network	Road network from TeleAtlas 2006 version	
ACTUAL FLOW	Flow direction and flow accumulation	EU Dem 100 m: https://land.copernicus.eu/pan-european/satellite-derived-products/eu-dem/eu-dem-v1-0-and-derived-products/eu-dem-v1.0?tab=download	
MONETARY VALUE	Water depth from flood hazard maps (all available return periods)	https://data.jrc.ec.europa.eu/collection/id-0054	
	Damage functions	Huizinga (2007)	
**Crop pollination**	Source: Vallecillo et al. 2018, page 33		
POLLINATION POTENTIAL	Land-use land cover data	CORINE Accounting Layers (https://www.eea.europa.eu/data-and-maps/data/corine-land-cover-accounting-layers#tab-european-data)	EU-26 (excluding Cyprus and Malta)
	Roads	Road network from TeleAtlas 2006 version	
	Climate data	Gridded Meteorological data from Agri4Cast (http://agri4cast.jrc.ec.europa.eu/DataPortal/SignIn.aspx?idResource=7&o=d) and E-OBS (http://www.ecad.eu/download/ensembles/download.php)	
	Species records	Bumblebee records from Atlas Hymenoptera (http://www.atlashymenoptera.net/)	
POLLINATION DEMAND	Pollination dependent crops	CAPRI data (https://www.capri-model.org/dokuwiki/doku.php)	
ACTUAL FLOW	Percentage of crop covered by SPA	CAPRI data (https://www.capri-model.org/dokuwiki/doku.php)	
	EUROSTAT [apro_cpsh1]	Eurostat dataset (https://appsso.eurostat.ec.europa.eu/nui/show.do?dataset=apro_cpsh1&lang=en)	
MONETARY VALUE	EUROSTAT [aact_uv01]	Eurostat dataset (https://appsso.eurostat.ec.europa.eu/nui/show.do?dataset=aact_uv01&lang=en)	

CULTURAL			
Nature-based recreation	Source: Vallecillo et al. 2018, page 11		
NATURE-BASED RECREATION POTENTIAL	Land-use land cover data	CORINE Accounting Layers (https://www.eea.europa.eu/data-and-maps/data/corine-land-cover-accounting-layers#tab-european-data)	Analysis of trends can only be done for member states of the EU in 2000 (Austria, Belgium, Germany, Denmark, Spain, Finland, France, Greece, Ireland, Italy, Luxembourg, Netherlands, Portugal, Sweden, United Kingdom)
	Protected areas	World database of Protected areas (https://www.iucn.org/theme/protected-areas/our-work/world-database-protected-areas)	
	Bathing Water Quality	State of Bathing water (https://www.eea.europa.eu/themes/water/status-and-monitoring/state-of-bathing-water/state/state-of-bathing-water-3)	
	Distance to Coast (sea and inland water bodies)	CORINE Accounting Layers (https://www.eea.europa.eu/data-and-maps/data/corine-land-cover-accounting-layers#tab-european-data)	
	Coastal geomorphology	EUROSION Coastal Erosion Layer (Eurosion 2005)	
	TeleAtlas	Road network from TeleAtlas 2006 version	
	Residential areas	CORINE Accounting Layers (https://www.eea.europa.eu/data-and-maps/data/corine-land-cover-accounting-layers#tab-european-data)	
DEMAND FOR NATURE-BASED RECREATION	Population	Global Human Settlement Layer (http://ghsl.jrc.ec.europa.eu/ghs_pop.php)	
ACTUAL FLOW	Local administrative units	http://ec.europa.eu/eurostat/web/nuts/local-administrative-units	
	Mobility function	UK-Monitor of Engagement with the Natural Environment (https://www.gov.uk/government/collections/monitor-of-engagement-with-the-natural-environment-survey-purpose-and-results)	
MONETARY VALUE	Application of the Zonal Travel Cost Method	Technical details on Appendix I of JRC report (http://publications.jrc.ec.europa.eu/repository/handle/JRC110321)	
EEA (2018) National emissions reported to the UNFCCC and to the EU Greenhouse Gas Monitoring Mechanism. Published by Eurostat (update 05/06/2018). Downloaded 06/06/2018. Retrieved from http://appsso.eurostat.ec.europa.eu/nui/show.do?lang=en&dataset=env_air_gge			
Huizinga, H.J. (2007) Flood damage functions for EU member states. Technical report, HKV Consultants. Implemented in the framework of the contract # 382441-F1SC awarded by the European Commission—Joint Research Centre			
OECD (2016) Effective Carbon Rates: Pricing CO2 through Taxes and Emissions Trading Systems. OECD Publishing, Paris. Retrieved from http://dx.doi.org/10.1787/9789264260115-en			
Pérez-Soba, M., Elbersen, B., Braat, L., Kempen, M., Wijngaart, R., Staritsky, I., Rega, C. and Paracchini, M.L. (2019) The emergy perspective: natural and anthropic energy flows in agricultural biomass production. JRC116274, Publications Office of the European Union, Luxembourg. Retrieved from http://publications.jrc.ec.europa.eu/repository/handle/JRC116274			
Vallecillo, S., La Notte, A., Kakoulaki, G., Kamberaj, J., Robert, N., Dottori, F., Feyen, L., Rega, C. and Maes, J. (2019a) Ecosystem services accounting. Part II-Pilot accounts for crop and timber provision, global climate regulation and flood control, EUR 29,731 EN, Publications Office of the European Union, Luxembourg. Retrieved from http://publications.jrc.ec.europa.eu/repository/handle/JRC116334			
Vallecillo, S., La Notte, A., Polce, C., Zulian, G., Alexandris, N., Ferrini S. and Maes, J. (2018) Ecosystem services accounting: Part I - Outdoor recreation and crop pollination, EUR 29,024 EN; Publications Office of the European Union, Luxembourg. Retrieved from http://publications.jrc.ec.europa.eu/repository/handle/JRC110321			

## Footnotes

1 http://ec.europa.eu/environment/nature/capital_accounting/index_en.htm

2 We here refer to provisioning, regulating and maintenance, and cultural.

3 It is also known as ‘supply’ or ‘capacity’ in the ecological literature; however, the use of this concept would be confusing with the actual flow presented in the supply table (Maes et al., 2013; Villamagna et al., 2013).

4 Government can not be used as synonym of society. In the case of Carbon Trading Schemes government is indeed responsible to manage the process but they are not the only receiver of the climate regulation ES which is directed to the global society.
